# Structural Health Monitoring of Railway Transition Zones Using Satellite Radar Data

**DOI:** 10.3390/s18020413

**Published:** 2018-01-31

**Authors:** Haoyu Wang, Ling Chang, Valeri Markine

**Affiliations:** Railway Section, Delft University of Technology, 2628CN Delft, The Netherlands; L.Chang@tudelft.nl (L.C.); V.L.Markine@tudelft.nl (V.M.)

**Keywords:** satellite InSAR, transition zones, railway, settlement, structural health inspection

## Abstract

Transition zones in railway tracks are locations with considerable changes in the rail-supporting structure. Typically, they are located near engineering structures, such as bridges, culverts and tunnels. In such locations, severe differential settlements often occur due to the different material properties and structure behavior. Without timely maintenance, the differential settlement may lead to the damage of track components and loss of passenger’s comfort. To ensure the safety of railway operations and reduce the maintenance costs, it is necessary to consecutively monitor the structural health condition of the transition zones in an economical manner and detect the changes at an early stage. However, using the current in situ monitoring of transition zones is hard to achieve this goal, because most in situ techniques (e.g., track-measuring coaches) are labor-consuming and usually not frequently performed (approximately twice a year in the Netherlands). To tackle the limitations of the in situ techniques, a Satellite Synthetic Aperture Radar (InSAR) system is presented in this paper, which provides a potential solution for a consecutive structural health monitoring of transition zones with bi-/tri-weekly data update and mm-level precision. To demonstrate the feasibility of the InSAR system for monitoring transition zones, a transition zone is tested. The results show that the differential settlement in the transition zone and the settlement rate can be observed and detected by the InSAR measurements. Moreover, the InSAR results are cross-validated against measurements obtained using a measuring coach and a Digital Image Correlation (DIC) device. The results of the three measuring techniques show a good correlation, which proves the applicability of InSAR for the structural health monitoring of transition zones in railway track.

## 1. Introduction

**Figure 1:**
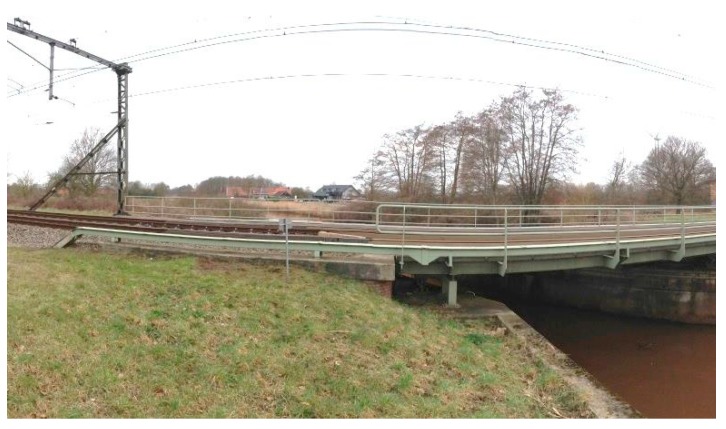
A typical track transition zone in the Netherlands.

Transition zones in railway track network are locations with considerable changes in the vertical support structures, which are located near engineering structures, such as bridges, culverts and tunnels [1]. A typical embankment-bridge transition zone is shown in Figure 1. Due to the difference in the stiffness and the settlement characteristics as well as the geotechnical and construction issues [2,3,4,5,6,7], the track geometry always degrades faster in transition zones than in open tracks, which causes significant irregularity (dips) in the track geometry. Such geometry irregularities may trigger considerable wheel-rail interaction forces, resulting in damage to track components, affecting the passenger’s comfort, and even causing derailment [3,8,9,10,11]. As a result, the maintenance of the track geometry in the transition zones requires substantial efforts. For example, in the Netherlands, the track maintenance in transition zones is performed up to 4–8 times more than on free tracks [12,13]. In the US $200 million is spent annually to maintain the transition zones, while in Europe about €97 million is spent [14,15].

To ensure the safety of railway operations and the reduction of maintenance costs, it is necessary to inspect the structural health condition of transition zones at a high frequency. Structural Health Monitoring (SHM) system would allow the detection of potentially dangerous situations at an early stage to provide an efficient way to extend the operational life of railway structures, while not interfering with train operations [16]. The widely used SHM systems are often impedance-based, which exploit the relationship between the electrical impedance of piezotransducers (PZT) and the mechanical impedance of the host structure to which the PZTs are bonded or embedded. The results, which are the inverse of the electrical impedance, are functions of the stiffness, mass, damping of the host structure; or length, width, thickness, orientation, and mass of the PZTs. Therefore, the structural damage can be detected from the variation of the monitoring results [17,18,19,20,21]. The current monitoring for transition zones is mainly aimed at observing the rail deflection performed by track-measuring coaches at the frequency of twice a year and rail/sleeper displacements at a higher frequency but only for individual case studies [22,23,24]. Both these SHM systems perform at a low frequency, which are mainly due to the inspection expense.

Therefore, this paper proposes a contactless SHM system that uses satellite SAR (Synthetic Aperture Radar) measurements to complement the current in situ measurements. The satellite SAR measurements are obtained on a bi-/tri-weekly basis, and with high precision (at millimeter level) [25,26]. By providing a stack of SAR measurements over a certain period, the settlement behavior of railway infrastructures can be retrieved using satellite radar interferometric (Synthetic Aperture Radar Interferometry, referred as InSAR) techniques [27,28,29]. InSAR has been successfully applied to the long-term railway monitoring in [27,30,31,32].

To demonstrate the InSAR feasibility and its potential for the structural health monitoring in transition zones, a transition zone is measured and analyzed using the InSAR techniques. Further, the InSAR results are cross-validated using the measurements from a measuring coach and a DIC-device. The paper is organized as follows. The settlement mechanism of the tracks in transition zones is described in Section 2. The measuring principle of the InSAR techniques and the conventional monitoring systems, including the measuring coach and the Digital Image Correlation (DIC)-based device is illustrated in Section 3. Section 4 introduces the testing site and the data collection using the monitoring systems. The settlement history of the transition zone measured using the InSAR techniques is analyzed in Section 5. To verify the InSAR results, the transition zone is also evaluated by other two validated measuring systems (the measuring coach and the DIC-based device). The relationship between the three measurement systems is discussed in Section 6. The conclusions are eventually drawn in Section 7.

## 2. Settlement Mechanism in Transition Zones

Since the vertical track stiffness determines the rail deflection due to passing trains, the large abrupt change in the vertical track stiffness in transition zones results in a rapid change in the elevation of wheels [8]. In other words, the wheels “drop down” when trains move from the engineering structure to ballast track, while the wheels are “lifted up” when trains move from the ballast track to engineering structure. In addition to the rail deflection caused by the different track stiffness, the ballast track always settles more as compared to engineering structures. According to the field measurements and laboratory tests [33,34,35,36,37,38,39], the ballast track is rapidly compacted immediately after construction or maintenance. Moreover, there are also some geotechnical, construction and maintenance issues, such as inferior quality of used materials, poor drainage conditions, inadequate compaction and consolidation of embankment or backfill [1] (before the construction of the bridge, the embankment is often excavated and refilled afterwards with the backfill). The three factors, including the stiffness variation, the different settlement behavior, and the geotechnical issues, lead to the differential settlement in transition zones. In [38], the differential track settlement up to 7 mm appeared in the transition zone after half-year operation. In another transition zone [1], the settlement was estimated up to 10 mm after the 10-month operation.

**Figure 2:**
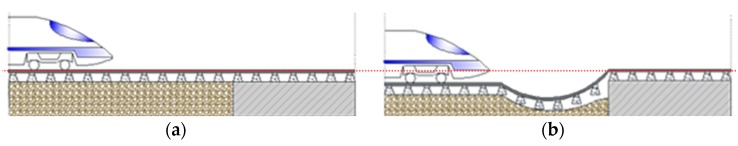
Schematic diagram of differential settlement in transition zone: (**a**) immediately after construction or maintenance; (**b**) after a few months of operation.

As a result, the wheel-rail interaction forces in transition zones are significantly amplified [6,8,40,41,42,43,44]. The analytical results in [40] showed that the wheel-rail interaction forces in the transition zone between the ballast track and slab track were increased up to 54% due to the differential settlement. Similarly, the increase of the wheel-rail interaction forces in the transition between the embankment and the bridge was up to 85% by the differential settlement [41]. The increased wheel-rail interaction forces ultimately lead to the accelerated deterioration of the vertical track geometry, as shown in Figure 2.

As shown by the “dip” in Figure 2b, the extra settlement of the tracks near the engineering structure has been confirmed by [1,3,38,45], which typically appears shortly after the installation/renewal of the track. A survey of the performance of track transition zones [2] reveals that 51% of the track transition zones described in that study have experienced such a settlement.

The affected zones located next to engineering structures often have various lengths, e.g., 4 m in [23,24], 7 m in [13,22], and 30 m in [3]. In [46], the following formula for the length of the affected zone L is suggested:

(1) $$L=\sqrt{1.6\times \Delta h\times V_{e}^{2}}$$

where Δh is the height difference of track in the transition zone (m); $V_{e}$ is the design speed (km/h). Assuming Δh is 0.01 m and $V_{e}$ is 160 km/h, the length of the affected zone L is approximately 20 m.

## 3. Condition Monitoring of Transition Zones

The current structural health-monitoring measurements for transition zones are mainly performed by measuring coaches, leveling instruments, and linear variable differential transformers (LVDT). Leveling instruments are the optical instruments used to establish or verify the points in the same horizontal plane, which were used in [3,22,47]. LVDTs convert a position or linear displacement from a mechanical reference into a proportional electrical signal containing phase and amplitude information, which was used among others in [38]. A complete introduction to SHM methods used on civil infrastructures can refer to [17]. The most widely used method is the track inspection performed by the measuring coach, which is presented in Section 3.1. Moreover, a detailed measurement method using DIC devices is explained in Section 3.2. The measuring principle of InSAR is provided in Section 3.3. It should be noted that the measuring principles of the three systems are different but related. The relationship between them is discussed in Section 6.

### 3.1. Measuring Coach

**Figure 3:**
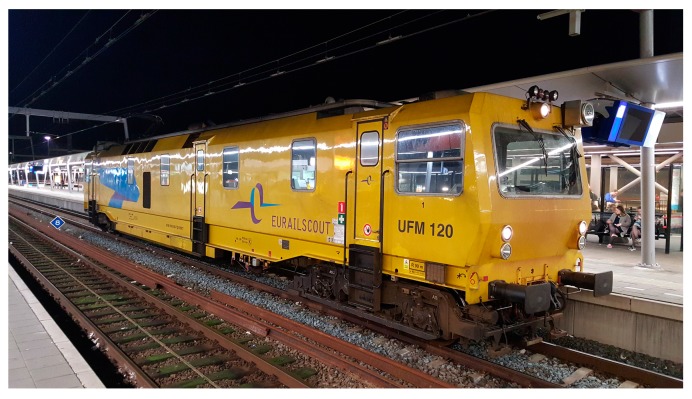
EURAILSCOUT UFM120 measuring coach [48].

In most countries, track geometry is mainly measured by the inertial measuring system of measuring coaches. For example, in Japan, the condition monitoring has been accomplished using measuring coaches since 1964. The measurement results are compared at a common point of reference. Precise allocation of a test result to a rail is possible via accurate DGPS (Differential Global Positioning System) location reference. For example, the EURAILSCOUT UFM120 measuring coach has been used since 1999 in the Netherlands, as shown in Figure 3 [48]. The car is self-propelled by a diesel engine. It operates in both directions with a maximum speed of 120 km/h. The basic technical information of the UFM120 is given in Table 1 [5].

The inertial measuring system consists of three high-precision gyro systems and accelerometers for all three spatial directions *x*, *y*, *z*. The position of measuring coaches is determined by means of double integration of the acceleration measurements. The track position is then measured in relation to the resulting coach position by using relative displacement transducers (optical track-measuring system). Assuming that the track settlement on engineering structures is negligible, the settlement of ballast tracks can be calculated by using the track on engineering structure as a reference.

This geometry measuring system is on the front side of car bodies, positioned between axles. Track geometry is measured at every 0.25 m, stored in onboard computer systems processed and printed immediately during the ride. All the measured data can also be plotted as a graphical representation, as well as be exported to the external data medium [5]. The track geometry signals are produced in three wavebands, which are 3–25 m (D1), 25–70 m (D2) and 70–150 m (D3) and the precision is 0.5 mm [49].

The advantage of measuring coaches is that it can measure multiple parameters at the same time, including track geometry, track videos/photos, and acceleration of bogies/car bodies. However, the cost of performing the measuring coach measurement is high so that its frequency is relatively low, for instance, twice per year in the Netherlands.

### 3.2. DIC Devices

DIC is an optical method using tracking and image registration techniques for accurate measurements in images. A reference picture is captured before loading, and a series of pictures are taken subsequently during the loading process. The images are analyzed using a numerical matching technique to identify the most similar patterns in the subsequent images, which is based on the assumptions that the pattern is approximately constant between successive images and the local textural information is unique. The matching algorithm compares the image subsets in the reference image with the image subsets in the current image [50]. Matching criteria are available such as in [51,52]. The method can continuously record of in-plane displacements of targets without any contact, which excludes any interference between the measured surface and the measuring device [53,54,55].

**Figure 4:**
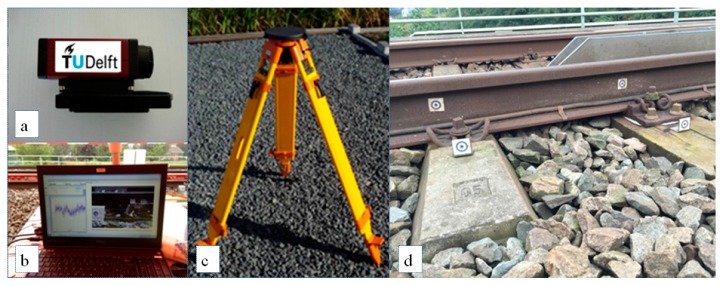
A DIC-based device: (**a**) a high-speed camera; (**b**) processing system; (**c**) a tripod; (**d**) targets.

The DIC devices have been used to monitor the displacement of rails [24], displacement of sleepers [56,57,58], and strain of rails [59] during train passages. The DIC system can be even mounted on railcars to perform an interrogation of sleepers while moving [16,60]. A DIC device often consists of high-resolution digital cameras that record the displacement of targets, and post-processors that analyze the changes in images. The DIC device used in this study is shown in Figure 4. This device was successfully used in railway applications such as monitoring turnout crossings [61,62].

DIC devices can measure detailed dynamic responses of the tracks at specific locations in transition zones. However, the cost of performing DIC measurements is also at a high level since it needs the manual operation. Currently, the condition inspection of transition zones using DIC devices is only performed occasionally and locally for the purpose of research.

### 3.3. Satellite Radar Interferometry

InSAR techniques have been widely applied in monitoring “natural” geophysical processes, and recently, they have been successfully deployed for structural health monitoring of infrastructure, such as railway, buildings and bridges [27,31,63,64].

**Figure 5:**
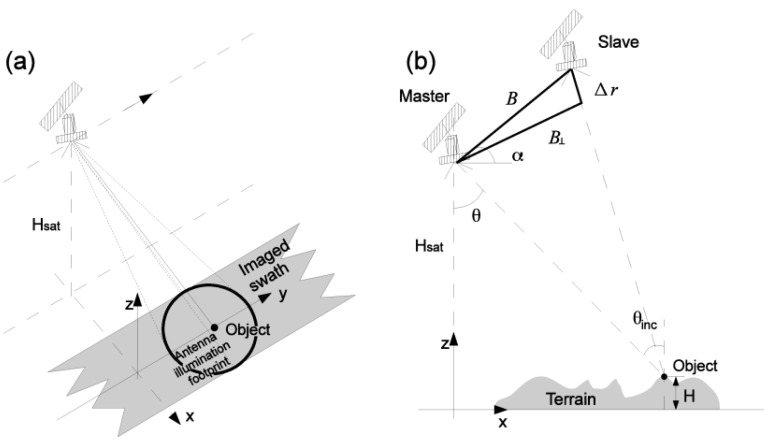
(**a**) SAR and (**b**) repeat-pass InSAR imaging geometry, adapted from [29]. (**a**) The satellite at height $H_{sat}$ moves along the azimuth direction for y axis and the direction of the main lobe of microwave pulse is nominated as the range direction for x axis. The area circled by black indicates the footprint of a single pulse. (**b**) Two SAR sensors, master and slave are at a distance by a baseline B. The perpendicular baseline $B_{⊥}$ is defined by the effective distance between master and slave sensors, measured perpendicular to the look direction. The angle between B and horizontal plane is defined as α. The look angle θ is the angle with which the radar looks at the surface. The incidence angle $\theta_{inc}$ is defined with regard to the ellipsoid. The height of the object is H with regard to a reference surface.

InSAR uses two or more satellite radar measurements (i.e., SAR images, acquired by SAR satellites) of the same area to extract ground deformation remotely and periodically, with millimeter-level precision. A SAR satellite operates using a side-looking geometry and illuminates a swath parallel to the satellite’s nadir track by transmitting a series of radar pulses from a fixed antenna [65], see Figure 5a. The ground targets with a strong scattering reflection, e.g., rail bars, can be well observed using SAR satellites. It should be noted that the observability for ground targets may be degraded when the ground targets are temporarily covered by ice, snow, or water. Therefore, the accuracy of railway measurements may be reduced in the extreme weather. However, its effect is not discussed in the paper since such weather is very rare in the Netherlands.

When two (or more) SAR images, acquired at the same time from a different orbital track or acquired at the separated time but imaging the same area, are available, the temporal evolution of ground targets can be retrieved by the InSAR techniques. For instance, the two SAR images are called the master image and slave image; see Figure 5b. The slave image must be co-registered and resampled with regard to the geometry of the master image. The interferometric phase is derived from the pixel-by-pixel multiplication with the conjugate complex of a pixel, in the master and slave image. Such complex multiplication yields a value $\phi^{w}\in \left[-\pi ,\;\pi \right)$ [rad] that referred to as the “wrapped”, or relative, interferometric phase. If the “absolute” phase ϕ∈ℝ (ℝ is the set of real numbers) were observed, it would be directly related to the slant range difference ∆r between master and slave (see Figure 5b), as in

(2) $$E\left\{\phi \right\}=-\frac{4\pi }{\lambda }∆r,$$

where E{.} expresses the expectation operator. Note that Equation (2) holds for the repeat-pass configuration where the two SAR images are acquired at different times. However, the absolute interferometric phase ϕ cannot straightforwardly be measured, due to the fact that the interferometric observed phase $\phi^{w}$. Instead, a relative phase, modulo 2π radians, expressed as W{φ}=mod{φ+π, 2π}−π is measured, where W{.} is the wrapping operator.

The absolute interferometric phase ϕ can be decomposed into four components that are surface topography, temporal displacement, atmospheric delay, and noise,

(3) $$\phi^{w}=W\left\{\phi \right\}=W\left\{\phi_{topo}+\phi_{defo}+\phi_{atmo}+\phi_{noise}\right\},$$

where
$\phi_{\mathrm{topo}}=-\frac{4\pi }{\lambda }\frac{B}{\rho \sin\theta }H$The topographic phase caused by the surface geometry, ρ is the slant range, H is the height above a reference surface$\phi_{\mathrm{defo}}=-\frac{4\pi }{\lambda }∆r$The kinematic phase due to surface or object displacement$\phi_{\mathrm{atmo}}$The phase contribution from the atmospheric signal delay$\phi_{\mathrm{noise}}$Other random additive noise.

It is of importance to properly unwrap the interferometric phase and mitigate the atmospheric and noise influence, in order to generate a high-precision deformation component. Therefore, the persistent scattered interferometric (PSI) method [25,66], using a stack of SAR images, is further proposed. During PSI processing, the temporal behavior of the ground targets with strong reflection, high coherence, stable phase values over time, can be observed. It is worthwhile to mention that all the satellite radar measurements are relative with regard to a reference epoch and reference point. By comparing the spatial coordinates of the unloaded tracks to the reference point, the increase of the track settlement at various locations can be obtained. Moreover, the high growth rate of the settlement indicates the fast degradation of the track, which suggests the track condition is poor, and vice versa. For instance, the authors in [20] demonstrates that the settlement behavior and seasonal deformation of the track can be well measured in the Himalaya region. In [18], the long-term settlement of a railway in the Netherlands measured by InSAR was validated against the measurement data of measuring coaches.

Comparing to the measuring coaches and DIC devices, which will be introduced in Section 3.2 and Section 3.3, SAR satellites bring higher frequent measurements, while the precision of the satellite measurements is at millimeter level, which is relatively low, and the spatial resolution/sampling of the satellite measurements is at meter level, which is larger than the measuring coach (0.25 m).

## 4. Test Site and Data Description

**Figure 6:**
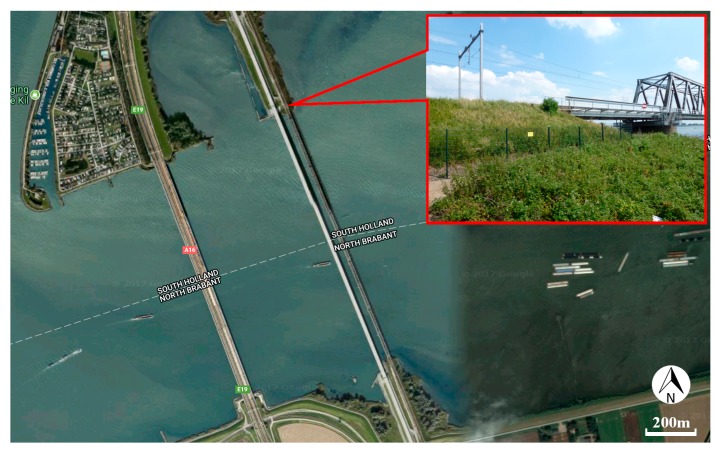
Photograph of the transition zone.

To study the capability of InSAR application on the structural health inspection of transition zones, a transition zone is analyzed. The settlement results for the transition zone derived from SAR data processing are compared with the results of the measuring coach and the DIC device performed on the same transition zone (as introduced in Section 3). The transition zone consists of the tracks on the steel bridge and embankment (located in Moerdijk, The Netherlands) as shown in Figure 6. The track on the embankment is the ballast track, while the track on the bridge is the embedded rail system.

The track settlement in the transition zone was measured by the SAR satellites. 153 TerraSAR-X satellite images (30 × 50 km) were acquired between April 2009 and October 2015, in descending orbit. The wavelength of the on-board radar sensor is 3.1 cm. The PSI method is used to process the SAR data. The measuring coach is EURAILSCOUT UMF 120 as described in Section 3.1. The track alignment was measured in April 2015. The signal was produced in 3–70 m (D1D2) wavebands. The DIC device shown in Figure 4 was employed to measure the vertical displacements of the rails at some locations in the transition zone. The measurement was performed in April 2015, when more than 30 train passages were recorded (sampling frequency 78 Hz). The key parameters of the three systems are summarized in Table 2.

## 5. Measurement Results

In this section, the InSAR measurement results are first discussed. Then the cross-validation is performed using the two in situ measurements from the measuring coach and the DIC devices.

**Figure 7:**
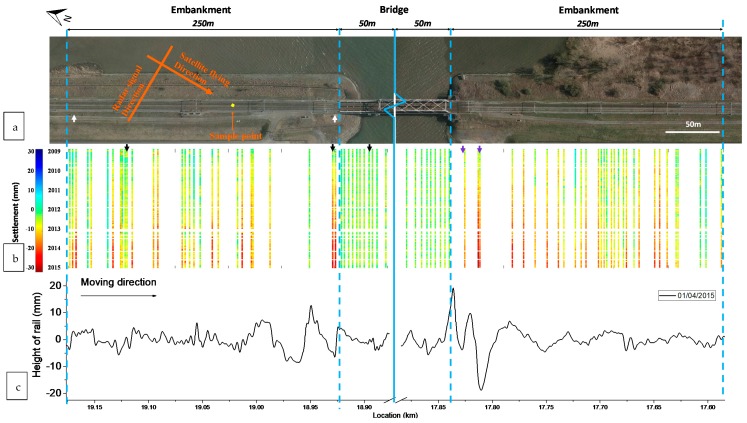
Measurement results of the transition zone: (**a**) Top-view photograph; (**b**) Settlement profile measured the satellite system; (**c**) Alignment measured by the measuring coach. The middle parts of the bridge are abbreviated. The yellow square indicates the sampling resolution (3 × 3 m). The satellite flying direction and radar signal direction are indicated. Write arrows are the locations where DIC measurements were performed. Black arrows are the points collected in Figure 8. Purple arrows are the locations of the expansion joints shown in Figure 11.

The track settlement measured by the TerraSAR-X satellites in the transition zone is shown in Figure 7b, along with the photograph of the transition zone (Figure 7a) and track alignment measured by the measuring coach (Figure 7c). The sampling resolution is indicated by the yellow square in Figure 7a, as well as the satellite flying direction and radar signal direction. Note that since the image resolution of the TerraSAR-X satellite is 3 m, the double tracks are considered as one. The satellite flying direction and radar signal direction are indicated as well. As SAR satellites can only observe the ground targets with a strong scattering reflection (mentioned in Section 3.3), the ground targets with a poor scattering reflection over this railway segment cannot be detected. Therefore, the spatial sampling is not always 3 m, and the distance between the adjacent SAR measurement points shown in Figure 7b varies.

As shown in Figure 7, the settlement on the embankment and the settlement on the bridge are so different that the bridge can be clearly distinguished from the embankment. The settlement of the track on the bridge is relatively stable with marginal fluctuations. On the contrary, the settlement of the track on the embankment increases over time. The increase of the settlement varies from different locations, i.e., the track settles more at the location close to the bridge and less at the location far from the bridge, which is in agreement with the findings from other field measurements in [3,24,38]. 

**Figure 8:**
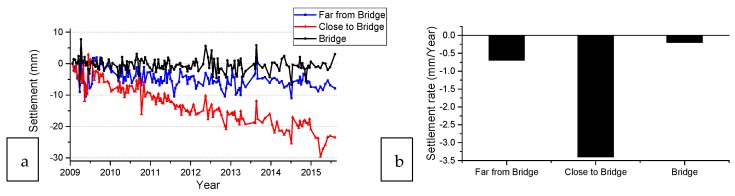
Settlement history of the typical points in the transition zones: (**a**) Settlement history curve; (**b**) Settlement rate.

To study the track settlement in different regions of the transition zone, the InSAR measurements in three representative points of the transition zone are further analyzed, including one point from the ballast track close to the bridge, one point far from the bridge and one point the on the bridge (indicated by the black arrows in Figure 7). The settlement histories of points and their rate are shown in Figure 8. Figure 8 clearly shows that track close to the bridge settles much faster than that farther from the bridge, while the settlement on the bridge remains stable. This proves that InSAR can measure the settlement rate in transition zones in this case, which can provide insightful information to understand the degradation process of the transition zones. It should be noted that the feasibility and the quality of InSAR-based structural health monitoring over transition zones may differ in other cases, due to e.g., the track orientation of a transition zone, satellite viewing geometry and satellite data availability. In addition, sufficient persistent scatters (PS)s are required in the transition zones in order to measure the condition. It can be insured either by increasing the resolution of the satellite or by installing an object (e.g., a pile) in the transition zones.

As shown in Figure 7, the fluctuation of the track alignment is significantly amplified near the bridge. On the contrary, the track irregularities are much smaller on the bridge and the embankment farther from the bridge. The results of the measuring coach (see Figure 7c) correlate very well with the results of the InSAR since the locations with the large fluctuations of track alignment are mostly the locations with the large settlement. It is reasonable because the differential settlement leads to the poor supporting condition of rails and sleepers, which can be observed as track irregularities. It should be noted that the track irregularities are not only caused by the differential settlement, but also by rail defects. This is the reason for the small discrepancies between the settlement and the track alignment.

**Figure 9:**
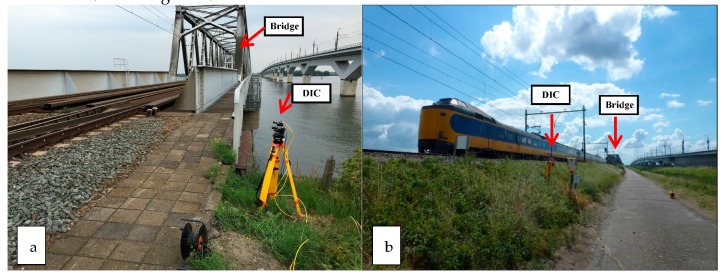
Measuring locations of the dynamic displacements of rail are measured by the DIC device: (**a**) the location close to the bridge, (**b**) the location far from the bridge.

**Figure 10:**
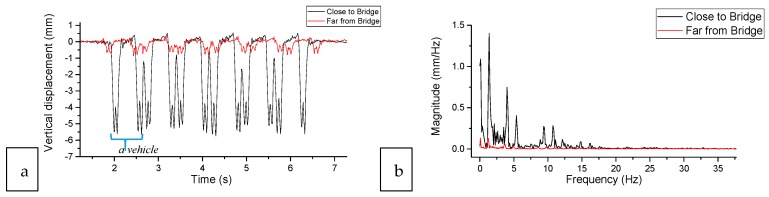
Vertical displacements of rail at 1.5 m and 4.5 m of the transition: (**a**) in the time domain, (**b**) in the frequency domain.

The dynamic displacements of rail at two typical locations near the bridge were measured by the DIC device. One location is close to the bridge (1.5 m) and the other is far from the bridge (240 m), as indicated by the two white arrows in Figure 7a and shown in Figure 9. The measurement results are shown in Figure 10.

Figure 10a shows the time curve of the vertical rail displacement. The downward peaks are corresponding to axles (24 wheelsets). The close two peaks belong to one bogie, e.g., the two peaks around 2 s. The relative distant two bogies are from one vehicle, e.g., the bogies at 2 s and 2.6 s (marked by the parentheses in Figure 10a). It can be seen in Figure 10, the amplitudes of the rail displacements at the two locations are of significant distinction, where the rail close to the bridge reaches 5.65 mm while the rail far from the bridge is only 0.87 mm. The large displacements of the rail close to the bridge imply the appearance of hanging sleepers, which confirms the differential settlement in the transition zone. Therefore, the settlement results measured by the InSAR are again validated by comparison with the measurement results of the DIC measurement.

**Figure 11:**
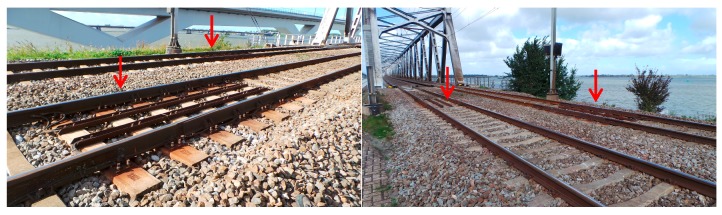
Expansion joints on the right side of the bridge.

It is remarkable to note that the transition on the right side (in Figure 7) of the bridge is in worse condition than that on the left side. Both the high settlement and track irregularity on the right side of the bridge indicate the track is in poor condition. Especially at 17.81 km, the settlement reaches 31 mm and the dip reaches 18.8 mm. After field observation, it is found that there are two expansion joints at the corresponding location, which probably causes the extra settlement and track irregularity. The expansion joints are shown in Figure 11 and their locations are indicated by the purple arrows in Figure 7. It shows that the InSAR data is also capable of locating the differential settlement caused by specific track components.

## 6. Discussion

The satellite, the measuring coach and the DIC measurement unveil the structural health condition of the transition zone. Since the measured objects from these three systems may differ, the relation between the three systems is discussed in this section.

**Figure 12:**
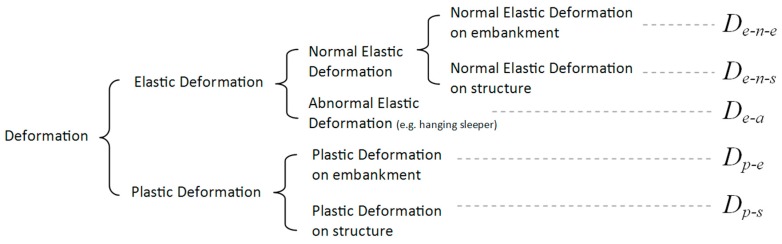
Deformation classification.

The track deformation is explained in Figure 12.

The normal elastic deformation is determined by the track stiffness, including the stiffness of rail, fastening system, and substructure. It can therefore be different between the track on the embankment and the track on the structure (e.g., embedded rail system on the concrete/steel bridge). The abnormal deformation represents the void under the sleepers (hanging sleepers). They often exist in transition zones [1,22,38], which are hanged by the bending stiffness of the rails and only appears when loaded. The plastic deformation is the track settlement.

As a result, the InSAR techniques can measure the settlement of the unloaded track, which is: $D_{p-e}$, $D_{p-s}$. The measuring coach can measure the alignment of loaded track. Considering track on the engineering structures as reference, the relative alignment can be obtained, which is: $\left(D_{e-n-e}-D_{e-n-s}\right)+D_{e-a}+\left(D_{p-e}-D_{p-s}\right)$. The DIC device can measure maximal displacement of track, which is: $D_{e-n-e}+D_{e-a}$, $D_{e-n-s}$.

In principle, the abnormal elastic deformation ($D_{e-a}$) is highly correlated with the plastic deformation ($D_{p-e}$). The local differential plastic deformation may lead to the abnormal elastic deformation, and continuous abnormal elastic deformation in turn causes the plastic deformation. For the transition zone in poor condition, the plastic deformation on embankment is always high as well as the abnormal elastic deformation and vice versa. It is evident in Figure 7 that the location where high settlement appears (in red) overlaps with the location where the relative track geometry has high amplification.

**Figure 13:**
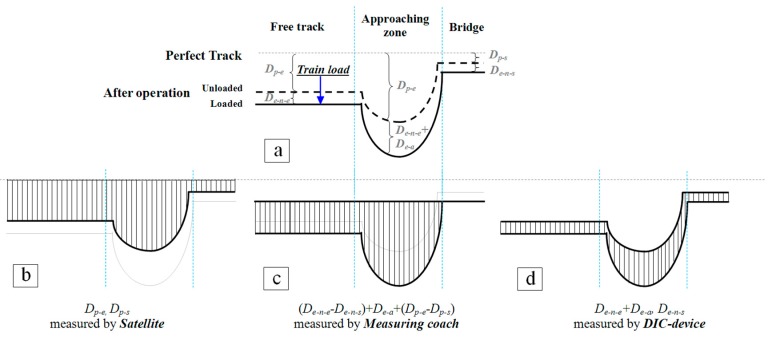
Schematic diagram of the measurement of the three systems. In (**a**), the unloaded track geometry after the operation is shown as the black dash line, while the loaded track geometry after the operation is shown as the solid black line. In (**b**–**d**), the deformation can be measured by the three systems is indicated as shade.

For an operated transition zone as shown in Figure 2b (with differential settlement), the measurements by SAR satellite, measuring coach and DIC, are shown in Figure 13, where the unloaded track geometry after the operation is shown as the black dash line and the loaded track geometry after the operation is shown as the solid black line. The deformation measured by the three systems is indicated as shade in Figure 13b–d.

Although the measured objects of the three systems are different, their results are inter-related. Therefore, they are capable of showing the health condition of the transition zones. Considering the cost efficiency, the satellite has an advantage in performing long-term health monitoring for transition zones at a high frequency.

## 7. Conclusions

This study demonstrates the feasibility of InSAR application on the SHM of transition zones in railway track. Measurements using the InSAR technique were performed on transition zones near a steel bridge. The results were cross-validated using the data from a measuring coach and a DIC device.

It has been proved that the settlement on the embankment and bridge are significantly different in the studied transition zone. The embankment and the bridge of the transition zone can be clearly distinguished in the satellite data. The track settles more in the locations close to the bridge and less in the locations far from the bridge. The settlement of the track on the bridge is relatively stable with marginal fluctuation.

The measurement results of the InSAR were compared with the results of two in situ monitoring systems, which are the measuring coach and the DIC-based device. The measuring coach measures the track alignment, and the DIC device measures the transient rail displacements. These measurements have a very good correlation.

The presented results have demonstrated that InSAR can be used in the health condition monitoring of transition zones. The InSAR system can monitor the health condition of the transition zones between the measurement interval of measuring coaches (half-year) and to provide guidance (suspect locations) for the local inspection or maintenance.

The questions needing further research are (1) how to ensure PSs in the measured transition zones and (2) how to guarantee the measurement accuracy in extreme weather.

## Figures and Tables

**Table 1:** Technical data of UMF120 measuring coach.

Parameter	Value
Date of construction	1998
Railway gauge	1.435 mm
Vehicle length over the buffer	23.000 mm
Centre casting distance	17.500 mm
Axle base	2.500 mm
Minimum turning radius in movement	90 m
Vehicle weight approximately	70 tons
Maximum drive speed	120 km/h
Maximum measurement speed	120 km/h

**Table 2:** Key parameters of the InSAR, the measuring coach and the DIC device.

Measurement Systems	Measured Object	Sampling Interval Along the Track (m)	Acquisition Dates
InSAR	Settlement	3 ^1^	2009-04~2015-10
Measuring coach	Track alignment	0.25	2015-04
DIC device	Displacement	0.6	2015-04

^1^ The acquired spatial resolution for the TerraSAR-X is 3 × 3 m. After post-processing, the spatial positioning precision along the track is 0.5 m [67].
